# Self-Supervised Denoising Image Filter Based on Recursive Deep Neural Network Structure

**DOI:** 10.3390/s21237827

**Published:** 2021-11-24

**Authors:** Changhee Kang, Sang-ug Kang

**Affiliations:** Department of Computer Science, Sangmyung University, Seoul 03016, Korea; 202032028@sangmyung.kr

**Keywords:** denoising filter, deep neural network, self-supervised learning, recursive training, raindrop removal

## Abstract

The purpose of this paper is to propose a novel noise removal method based on deep neural networks that can remove various types of noise without paired noisy and clean data. Because this type of filter generally has relatively poor performance, the proposed noise-to-blur-estimated clean (N2BeC) model introduces a stage-dependent loss function and a recursive learning stage for improved denoised image quality. The proposed loss function regularizes the existing loss function so that the proposed model can better learn image details. Moreover, the recursive learning stage provides the proposed model with an additional opportunity to learn image details. The overall deep neural network consists of three learning stages and three corresponding loss functions. We determine the essential hyperparameters via several simulations. Consequently, the proposed model showed more than 1 dB superior performance compared with the existing noise-to-blur model.

## 1. Introduction

Recently, cameras and sensors in autonomous vehicles and outdoor vision systems, such as closed-circuit televisions and dashboard cameras, are rapidly becoming important. Information obtained from visual and miscellaneous sensors should be as accurate as possible, because erroneous information can compromise both safety and property. However, the internal process of obtaining an image from a real scene using a camera is very complicated and is always accompanied by noise for various reasons. Since the shape and pattern of noise are random and unpredictable, it is difficult to design an appropriate denoising filter. Sometimes noise is caused by the external environment rather than the camera itself, including raindrops, snowflakes and even captions in images. So, various deep neural network approaches [1,2,3,4,5] have been proposed to remove such environmental noises.

There are two noise removal approaches, hand-crafted and deep neural network approaches. First, hand-crafted approaches use various image features to remove noise. Buades et al. [6] utilized the fact that natural images often exhibit repetitive local patterns and many similar regions throughout the image. Therefore, similarity can be calculated by calculating the L2 distance between the kernel region and any region of an image. Then, the filtered value is obtained by computing the weighted average of similar regions, where the weights are determined based on the similarity. Some transform-based methods have been proposed by assuming that a clean image is sparsely represented in a transform domain [7,8,9]. However, various types of images cannot be guaranteed to be well sparsely represented with a single transformation. Elad et al. [7] proposed a dictionary learning method. In this context, the dictionary is a collection of basic elements that can represent an image as their linear combination. The dictionary is updated and improved using the k-singular value decomposition (K-SVD) method for more appropriate sparse representations. Therefore, a denoised image can be estimated from the sparse representation using the final updated dictionary. However, this method consumes lots of computation to obtain the final updated dictionary. Inspired by the similarity concept used in the literature [6], Dabov et al. [8] proposed an advanced sparse representation method. Sparse representations are extracted from high similarity image regions instead of the entire image region, achieving approximately 0.3 dB higher denoised image quality than the method in [7]. Gu et al. [9] suggested weighted singular values to improve the SVD method and showed 0.3 dB better performance than the method in [8]. These methods typically require a high computational load to obtain denoised images and have performance limitations for unknown or variable noises, leading to the following deep neural network approaches. Second, some deep neural network methods have been proposed using state-of-the-art artificial intelligence technologies [10,11,12,13,14]. Zhang et al. [10] proposed a supervised learning model that can effectively remove Gaussian noise of various noise levels. A 20-layer convolution neural network (CNN) model is used with residual learning [15] and batch normalization [16]. Zhao et al. [17] improved the network model designed in [10] by combining temporary noises extracted from the last few network layers with the ground-truth noise. Usually, these supervised methods have a relatively good noise-filtering ability but require a dataset of noisy and clean image pairs, which is considerably difficult to obtain in the real-world. Thus, in most cases, such paired datasets are generated synthetically by adding synthetic noise to clean images.

On the other hand, self-supervised learning methods do not explicitly require the corresponding clean images, unlike supervised ones. Self-supervised methods use the ground-truth data created by slightly modifying or transforming the filter input data, which is not always easy and practical. Lehtinen et al. [12] proposed a noise-to-noise (N2N) learning method, where the ground-truth is a number of noisy images with noise exhibiting the same statistical characteristics as the original noise. The noise is supposed to be additive random noise with zero mean. If the L2 loss function is used, the deep neural network can learn a denoising ability even when multiple noisy images are used as ground-truth instead of a single clean image. The performance of the N2N method is somewhat inferior to those of the supervised learning methods. Additionally, creating target noisy images is occasionally difficult because the original and target noisy images have the same clean image, which is frequently impossible. To avoid this impractical situation, Krull et al. [13] designed a noise-to-void (N2V) technique, where ground-truth images are created by replacing pixels in the original noisy image with adjacent pixels. Since this method attempts to imitate the N2N, its performance is approximately 1.1 dB lower than that. For enhanced performance, a clever pixel replacement technique was suggested by Batson et al. [18], where ground-truth images are created by replacing pixel values with random numbers. This technique achieved a slightly better noise removal performance than the N2V [13]. Niu et al. [19] suggested another N2V model that creates ground-truth images by replacing pixels with the center pixel in a region with high similarity based on the concept defined in [6]. This method shows approximately a 0.5 dB better performance than the N2V [13]. Xu et al. [20] proposed a practical version of the N2N method using a doubly noisy image as the input image. A doubly noisy image is created by adding noise, which is statistically similar to the original noise, to the original noisy image. This approach achieved a performance similar to that of the N2N method at low noise levels but showed deteriorated performance when the noise increased above a certain level. Another method that does not require paired noisy and clean datasets was proposed by Lin et al. [14], called noise-to-blur (N2B) method. In this method, the target image is a blurred image filtered with a strong low-pass filter; the method almost eliminates the noise as well as the image details from the original noisy image. In this process, many types of noise, such as raindrops, snowflakes and dust, can be successfully removed along with image details, which means that the N2B method can remove various types of noise, unlike the N2N and N2V. However, it shows lower performance than the N2N method.

Generally, the deep neural network-based approaches can handle more diverse and complex types of noise than hand-crafted ones owing to their learning ability. However, supervised deep neural networks require a hard-to-generate dataset, despite their good noise removal ability. The N2V and N2B methods are not limited by dataset issues but show relatively low performance. In this paper, we propose a high-performance and self-supervised method without dataset problems by introducing a recursive learning stage and a stage-dependent objective function. The rest of the paper is structured as follows. In Section 2, the basic structure and concept of the N2B model are depicted. Section 3 describes the details of the proposed model. Section 4 describes dataset, experimental setup and simulation results. Finally, Section 5 concludes this paper.

## 2. Related Work

The proposed method is based on the N2B model [14] because it has less noise-type dependency and does not require paired noisy and clean datasets. But this model shows approximately 1.38 dB lower performance than the N2N method [12], which has both paired dataset and noise-type limitations. The N2B model consists of two concatenated subnetworks, the denoising and noise extraction subnetworks. In addition, the learning process consists of two stages, the initial and convergence stages.

### 2.1. Initial Stage

At this stage, the network roughly learns how to blur an image so that it can excessively remove noise and even some image details. An example of a blurred image is shown in Figure 2c. It is assumed that the noisy image *X* is the sum of a clean image *Y* and additive noise *n*, as expressed in Equation (1):

(1) X=Y+n

The relation between the denoising subnetwork $f_{D}$ and noise extraction subnetwork $f_{N}$ is expressed using Equation (2):

(2) $$f_{D}\left(X\right)=\hat{Y},\;f_{N}\left(X-f_{D}\left(X\right)\right)=\tilde{n}$$

where $\hat{Y}$ is an estimated clean image of *Y* and $\tilde{n}$ is an estimated noise of *n*. The entire network is trained with the initial objective function in Equation (3):

(3) $$L_{I}=\frac{1}{M}\sum_{i=1}^{M}\left|{\tilde{Y}}^{i}-{Y_{b}}^{i}\right|,\;\tilde{Y}=X-\tilde{n}$$

where *M* is the number of input noisy images and $Y_{b}$ represents the corresponding blurred image using a strong low-pass filter.

### 2.2. Convergence Stage

In the convergence stage, $f_{D}$ and $f_{N}$ explicitly start learning different roles, denoising and noise extraction, respectively. First, the synthesized noisy image $d=c+\tilde{n}$ and its corresponding target, clean image *c*, are used to further train the $f_{D}$ subnetwork with the following convergence objective function in Equation (4):

(4) $$L_{C}=\frac{1}{M}\sum_{i=1}^{M}\left|{\hat{c}}^{i}-c^{i}\right|,\;f_{D}\left(d\right)=\hat{c}$$

where $\hat{c}$ is the estimate of *c* denoised by $f_{D}$. Second, the $f_{N}$ subnetwork learns its noise extraction ability using Equation (3), which is the same loss function used in the initial stage. These two objective functions use the L1 distance metric because it is more effective for image restoration problems [21].

## 3. Proposed Noise-to-Blur-Estimated Clean (N2BeC) Model

**Figure 1:**
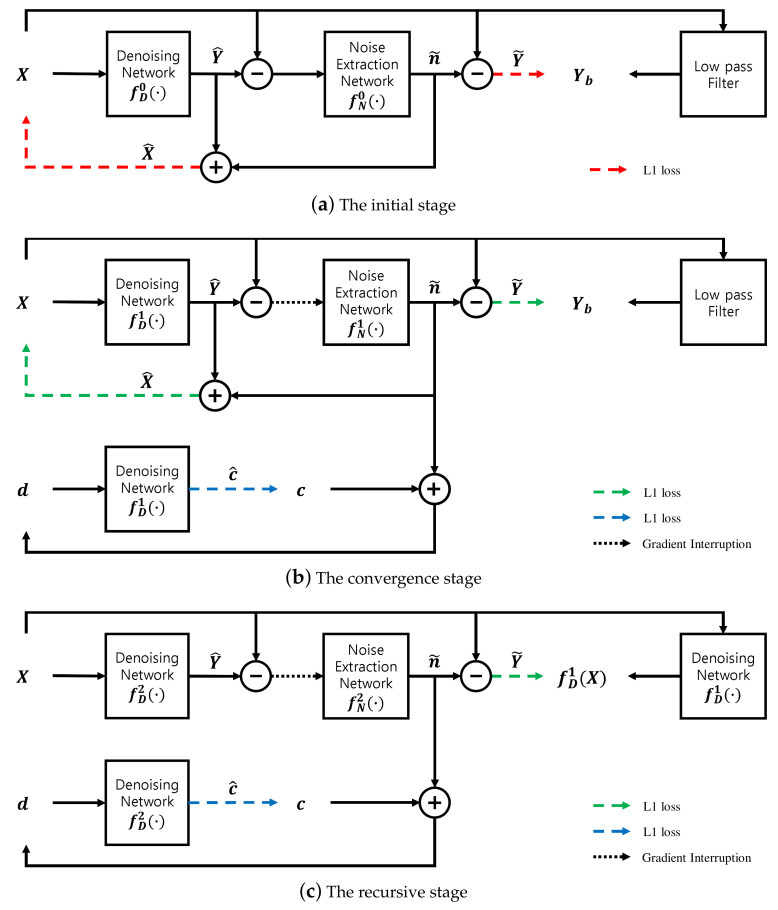
Block diagram of the proposed N2BeC method. (**a**) Initial, (**b**) convergence and (**c**) recursive learning stages.

The main aim of the proposed method is to improve denoising performance comparable to those of the N2N and supervised learning methods, while retaining the advantages of the N2B model. To achieve this goal, we propose a recursive learning method and a stage-dependent loss function. The overall diagram consists of initial, convergence and recursive learning stages, including three loss functions, as illustrated in Figure 1.

### 3.1. Recursive Learning Method

Since supervised learning methods usually show better performance due to the perfect ground-truth, the blurred image $Y_{b}$ can be replaced with the denoised image $f_{D}^{1}\left(X\right)$ after the completion of the initial and convergence stages. An important difference between the two ground-truth values $Y_{b}$ and $f_{D}^{1}\left(X\right)$ is whether the image details are retained. In $Y_{b}$, the noise is removed excessively, even including some image details, but some noise remains in $f_{D}^{1}\left(X\right)$ and most of the image details are retained. Both types of target images are easy to generate and complementary when used in time-series to train the network. Therefore, the N2B model can be enhanced if trained one more time using the denoised image of *X*. For this training, the recursive objective function is expressed by Equation (5). Hence, the recursive learning stage should be concatenated to the N2B model; this model is termed the N2B-estimated clean (N2BeC) model.

(5) $$L_{R}=\frac{1}{M}\sum_{i=1}^{M}\left|{\tilde{Y}}^{i}-{f_{D}^{1}\left(X\right)}^{i}\right|$$

### 3.2. Stage-Dependent Loss Function

In fact, a blurred image is unsuitable for ground truth, mainly because it lacks image details or high-frequency components. It effects the ability of a deep neural network to learn about image details, especially in the initial stage guided by Equation (3). This phenomenon continues in the convergence stage even though the effect is limited by the loss function, Equation (4). To compensate for the network’s ability to preserve image details while filtering noise, we use the input noisy image *X* as a supplementary target image in the initial and convergence stages, as shown in Figure 1a,b. The corresponding auxiliary objective function is expressed by Equation (6):

(6) $$L_{N}=\frac{1}{M}\sum_{i=1}^{M}\left|{\hat{X}}^{i}-X^{i}\right|,\;\hat{X}=\hat{Y}+\tilde{n}\;$$

This regularization is not helpful in the recursive learning stage, because the estimated clean image $f_{D}^{1}\left(X\right)$ that contains considerable image details is used as the target image. Finally, we propose novel objective functions by combining Equations (3)–(5) as follows:

(7) $$L_{NI}=L_{I}+\lambda \cdot L_{N}\;\left(in\;the\;initial\;stage\right)\;$$

(8) $$L_{NC}=L_{C}+L_{I}+\lambda \cdot L_{N}\;\left(in\;the\;convergence\;stage\right)\;$$

(9) $$L_{NR}=L_{C}+L_{R}\;\left(in\;the\;recursive\;stage\right)\;$$

## 4. Experimental Results

This section describes the dataset used in the experiments and compares the performance of the N2BeC model with hand-crafted, supervised and self-supervised methods.

### 4.1. Dataset Setup

**Figure 2:**
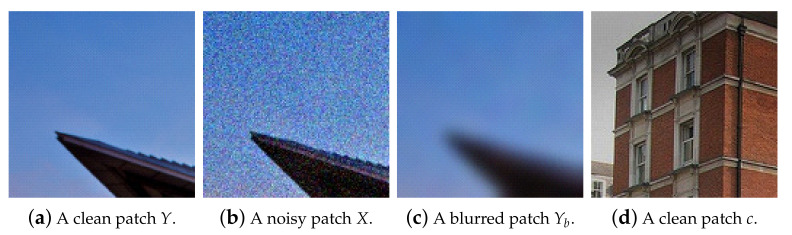
Example data: (**a**) a clean patch to generate noisy and blurred patches; (**b**) a noisy patch with Gaussian noise; (**c**) a blurred patch by a Gaussian filter with kernel size of 31; (**d**) a clean patch collected from the Internet.

The noisy image data *X* are generated by adding Gaussian noise *n* with various noise levels σ ranging from 0 to 50 to clean image data *Y*. We collected 4744 clean images from the Waterloo database [22] and used them to generate noisy images for training and validating the proposed network. In addition, the blurred image data $Y_{b}$ are created from *X* using a Gaussian filter with a kernel size of 31 to remove the noise added excessively. The actual noisy and blurred data for training and validation consisted of 10,000 non-overlapping patches with 128×128 size cropped from the original sized data, respectively. Some images were randomly obtained from the Internet and cropped to 128×128 sized patches. A total of 10,000 patches generated in this way were used for training as clean images *c*. Some examples are shown in Figure 2. For testing, 300 images from the BSD300 dataset [23] were used without resizing to reflect the real-world situation. The same dataset was used for simulating the N2N, N2B and the proposed methods in Table 1.

### 4.2. Network Structure and Training Period

The simple U-Net suggested in [14] was used as $f_{D}$. The network structure of $f_{N}$ had two 3×3 convolution layers with 32 feature maps, ReLU activation functions and the batch normalization method. The simple U-Net is a less-complexed version of the U-Net proposed in [24]. The batch size was set to 16 and the Adam optimizer [25] was applied. The network structure of the N2N is originally RED30 [26], but it was replaced by the simple U-Net for a fair comparison in these experiments. The N2B [14] and the proposed methods were trained for 50 and 950 epochs at the initial and convergence stages, respectively. The recursive learning stage was additionally trained for 100 epochs. The network was trained for 1100 epochs in total.

### 4.3. Performance Comparison

**Figure 3:**
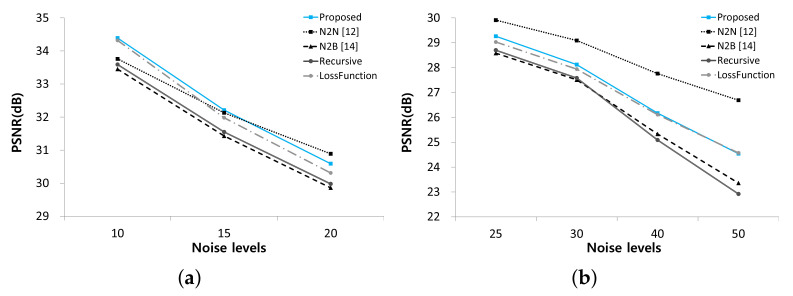
Comparison of denoising performance among N2N, N2B and proposed methods. (**a**) Noise levels from 10 to 20. (**b**) Noise levels from 25 to 50.

**Figure 4:**
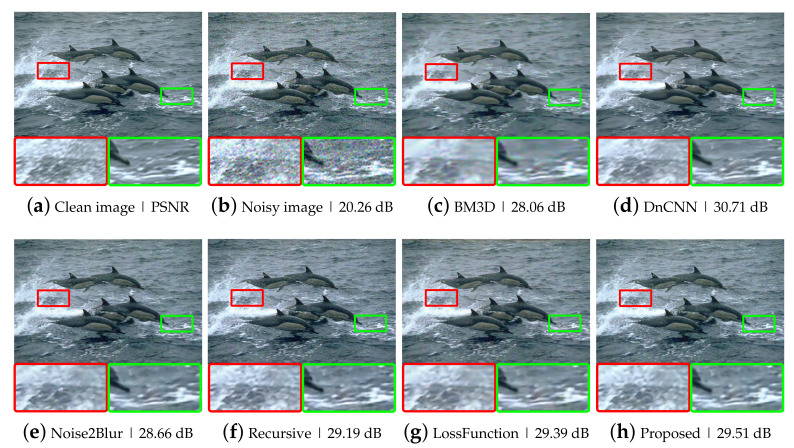
Example images of Gaussian denoising at σ=25: (**a**) a clean image; (**b**) a Gaussian noisy image; (**c**–**h**) example images of Gaussian denoising using various noise removal methods.

To evaluate the performance of the proposed method, we performed experiments using synthetic noisy images generated by adding different levels of Gaussian noise to the BSD300 dataset [23]. We conducted two experiments, one with fixed-level noise (Experiment I) and another with various level noise (Experiment II). The results of Experiment I are listed in Table 1 and Figure 3. The test results of the hand-crated method, BM3D [8] and the supervised deep neural network method, DnCNN [10], are provided to demonstrate the performance difference between substantially different methods, which are incomparable with the N2BeC. Since the recursive learning stage and the new loss function are introduced to improve the performance of the N2B model, we performed Recursive and LossFunction experiments to see how much each propose contributes to the overall performance improvement. The Recursive experiment consisted of three learning stages with Equations (3)–(5) in the initial, convergence and recursive stages, respectively; this way, it was possible to tell the effect of adding only the recursive learning stage to the N2B model. As a result, the Recursive experiment showed slightly better performance than the N2B at lower noise levels. The reason is that the target image $f_{D}^{1}\left(X\right)$ in the recursive learning stage contained little noise, which is a similar characteristic to $Y_{b}$, so that the network can consistently learn similar objectives in the entire stages. In contrast, the network performed slightly worse at high noise levels because the target image contained relatively much noise this time. Therefore, the training was inconsistent when switching the target image from the relatively noiseless $Y_{b}$ to the relatively noisy $f_{D}^{1}\left(X\right)$. Though, the overall performance was slightly better than that of the N2B model [14]. The LossFunction experiment consisted of the initial and convergence stages with the new loss functions, Equations (7) and (8), respectively. The simulation results indicate that the new loss function was effective at all noise levels, especially at high noise levels. The proposed N2BeC method is a combination of the Recursive and LossFunction experiments and shows synergistic performance. Some example images of denoising results at the noise level σ=25 are shown in Figure 4. Compared with the result of the N2B method, it can be seen that more image details remained in the proposed method. Compared with the N2N model, the proposed method had better performance at low noise levels, but the overall performance was low. However, it should be noted that the performance of N2N model was close to that of supervised methods in most cases.

**Figure 5:**
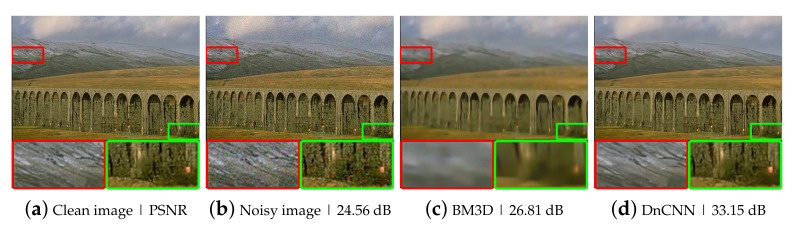

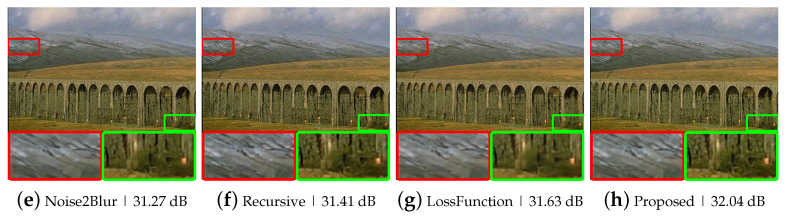
Example images of Gaussian denoising: (**a**) a clean image; (**b**) a Gaussian noisy image; (**c**–**h**) example images of Gaussian denoising using various noise removal methods.

Experiment II tested the denoising ability of the N2BeC method with noisy images at random noise levels between 0 and 50. The simulation results are listed in Table 2 and show a more than 1.05 dB better performance than the N2B model [14]. Figure 5 shows other example images for subjective quality comparison.

### 4.4. Ablation Study

**Optimal number of recursive learning stages.** An experiment was conducted to check if the performance could be continuously improved by repeating the recursive learning stage. It was assumed that the recursive learning stage was applied once in the N2BeC model, but not in the N2B model. When a second recursive learning stage was connected to the N2BeC network, the denoised $f_{D}^{2}\left(X\right)$ served as the target image. Similarly, $f_{D}^{3}\left(X\right)$ was the target image for a third one. The loss functions are expressed as Equations (10) and (11), respectively.

(10) $$L_{R2}=\frac{1}{M}\sum_{i=1}^{M}\left|{\tilde{Y}}^{i}-{f_{D}^{2}\left(X\right)}^{i}\right|$$

(11) $$L_{R3}=\frac{1}{M}\sum_{i=1}^{M}\left|{\tilde{Y}}^{i}-{f_{D}^{3}\left(X\right)}^{i}\right|$$

In this experiment, up to three recursive learning stages were repeated after the initial and convergence stages had finished using the Experiment II method in Section 4.3. The results are shown in Table 3. The optimal number of recursive learning stages was 1, which corresponds to the N2BeC model. This is because the trained denoising filter not only removes noise but also distorts the original content. If the noise removal filter is repeatedly applied over a certain number of times, the performance degradation due to content distortion is greater than the performance improvement by noise removal.

**Regularization factor of the new objective function.** We need to determine the regularization factor λ in Equations (7) and (8). Since $L_{N}$ is an auxiliary loss function, this hyperparameter should be greater than 0 and less than 1. Several simulations were performed using the Experiment II methodology in Section 4.3 to search for a proper value and the results are shown in Table 4. We found that the denoising performance was best at λ=0.1.

**Effect of $L_{N}$ in learning stages.** To investigate the effect of the new loss function in each stage, several combinations were tested. For example, $L_{N}$ was applied only to the initial stage using Equation (7) and not to the convergence and recursive learning stages using Equations (4) and (5), respectively. Another example is that $L_{N}$ was applied to all stages, even to the recursive learning stage, using Equations (7)–(9), respectively. The tested combination and the corresponding performances are listed in Table 5. Notably, the previous Recursive experiment is equivalent to the case where $L_{N}$ is not applied to any stage. According to the result, using $L_{N}$ only in the convergence step resulted in a slight performance decrease compared with the result obtained in the Recursive experiment. The initial stage’s only case improved the performance by 0.34 dB. However, the proposed case increased the performance by more than 1 dB, creating a synergy effect between the initial and convergence stages. Applying $L_{N}$ to the recursive learning stage degraded the denoising ability of the network.

**Recursive stage training.** The loss function of the recursive learning stage changed from $L_{NC}$ to $L_{NR}$, which is not smooth. In such a case, training the network more than an appropriate amount can, in turn, degrade the performance. Therefore, we investigated the proper amount of training in the recursive learning stage and the results are shown in Table 6. As a result of the search, it can be seen that the performance rather degraded after 100 epochs. Generally, if the loss function is not changed in the middle, the longer the training, the better or maintained the performance.

**Figure 6:**
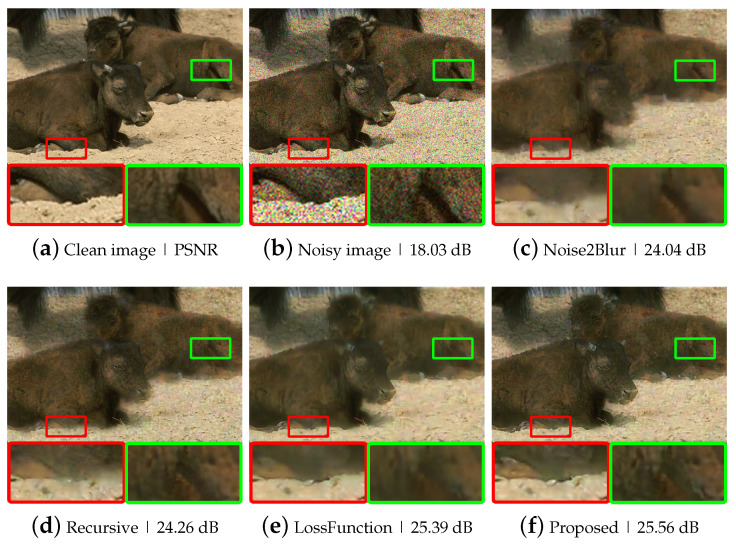
Example images of speckle denoising at v=0.1: (**a**) a clean image; (**b**) a speckle noisy image; (**c**–**f**) example images of speckle denoising using various noise removal methods.

**Figure 7:**
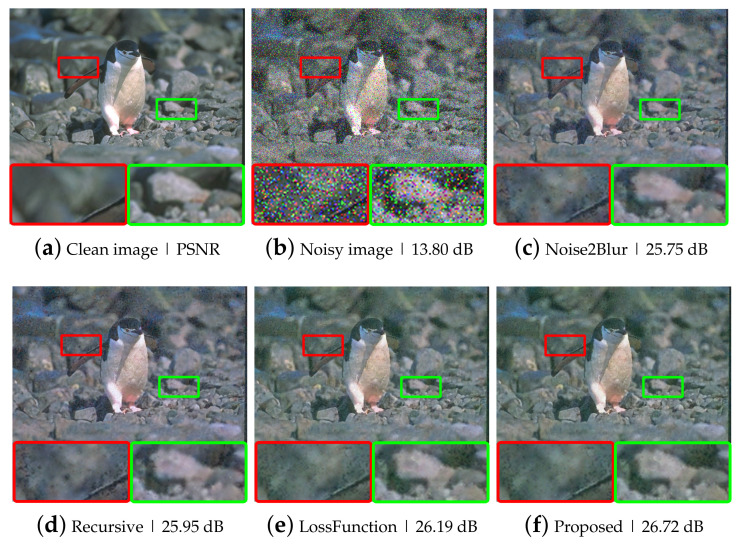
Example images of salt and pepper denoising at p=0.15: (**a**) a clean image; (**b**) a salt and pepper noisy image; (**c**–**f**) example images of salt and pepper denoising using various noise removal methods.

**Effectiveness for removing speckle, and salt and pepper noise.** In this auxiliary experiment, the Gaussian noise was replaced by salt and pepper noise and speckle noise, respectively. We used the same blur image generation method as in Section 4.1. For salt and pepper noise, the proposed network was trained with various noise probabilities ranging from p=0 to p=0.3 and tested with fixed noise probability p=0.15 and variable ones p∈[0,0.3]. For speckle noise, various noise variances *v* ranging from 0 to 0.2 were used for training and fixed, while varying noise variances were used for testing, as shown in Table 7. Some denoising examples are illustrated in Figure 6 and Figure 7. The proposed methods achieved higher performance in removing various types of noise than the N2B method, as shown in Table 7.

**Figure 8:**
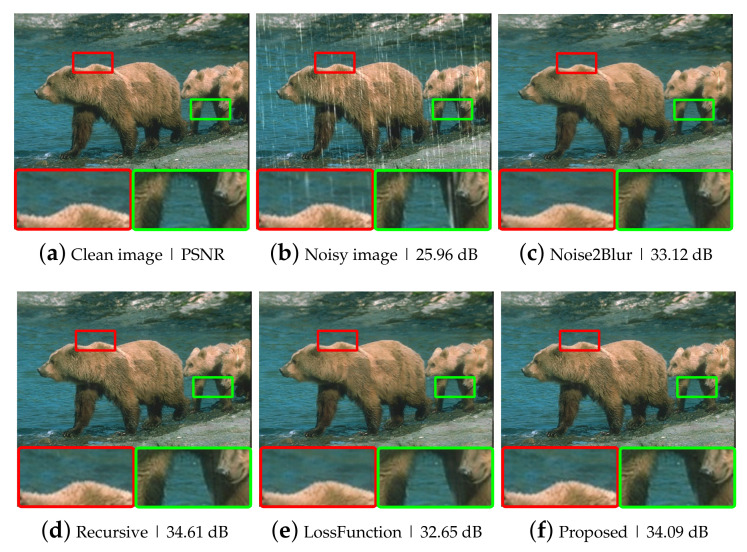
Example images of raindrops removal: (**a**) a clean image; (**b**) a raindrop noisy image; (**c**–**f**) example images of raindrops removal using various noise removal methods.

**Effectiveness for removing raindrops.** In this auxiliary experiment, the Gaussian noise was replaced by raindrops, which can be considered as a sort of noise. Since the raindrops were not removed enough with the Gaussian low pass filter, a median filter with a kernel size of 31 was used to make blur images. A total of 200 images from the Rain100L dataset [27] was used for training and 100 images for testing. Some examples of raindrop removal are displayed in Figure 8. Again, the proposed methods achieved higher performance in removing raindrops than the N2B method, as shown in Table 8.

## 5. Conclusions

Collecting only noisy data is easy and cheap. In this work, we suggest a novel denoising deep neural network model that does not require a noisy and clean data pair for ensuring the practicality of the proposed method. In addition, since the proposed N2BeC model is based on the N2B model, it can be extended to remove environmental noises such as raindrops, snowflakes and dust. Importantly, the noise removal performance is superior to those of the N2V and N2B models, which are real supervised methods. Therefore, the N2BeC model is not only practical and extendable but also has good performance due to the introduced recursive learning stage and stage-dependent loss functions. The multi-stage learning method using deep neural networks approaches the correct answer by giving more accurate hints as the stages progress. In this paper, the number of recursive stages is limited to two, but if the number can be increased in the future, it is expected that the performance can be further improved. In addition, it is possible to find an adaptive method that can be applied to more various types of noise.

## Figures and Tables

**Table 1:** Comparison of denoising performance for noisy image with fixed-level noise.

Methods	Noise Level (σ)	10	15	20	25	30	40	50
	Noisy Image Quality	28.25	24.80	22.37	20.50	19.00	16.69	14.97
BM3D [8]		33.15	30.91	29.38	28.21	27.27	25.77	24.56
DnCNN [10]		35.65	33.51	32.04	30.91	30.02	28.62	27.48
N2N [12]		33.76	32.13	30.89	29.91	29.09	27.76	26.69
N2B [14]		33.45	31.43	29.86	28.58	27.51	25.33	23.36
Recursive		33.59	31.55	29.98	28.70	27.58	25.09	22.92
LossFunction		34.32	31.98	30.31	29.03	27.94	26.11	24.57
Proposed		34.39	32.21	30.59	29.26	28.12	26.17	24.54

**Table 2:** Comparison of denoising performance for noisy image with various noise levels.

Noise Level	BM3D [8]	DnCNN [10]	N2N [12]	N2B [14]	Recur.	LossF.	Proposed
σ∈[0,50]	25.95	32.08	30.48	29.10	29.14	30.01	30.15

**Table 3:** Effects of successive recursive learning stages.

No. of Recursive Stages	0 (=N2B)	1 (=N2BeC)	2	3
quality (PSNR)	29.10	29.14	28.64	28.40

**Table 4:** Effects of regularization factor of the proposed objective function.

λ	0.01	0.05	0.1	0.2	0.5	1.0
quality (PSNR)	29.80	29.87	30.01	28.96	25.01	23.99

**Table 5:** Performance comparison according to the use of the proposed objective function.

Learning Stage			Image Quality (PSNR)
Initial	Convergence	Recursive	
×	×	×	29.14
∘	×	×	29.48
×	∘	×	29.11
×	×	∘	28.56
∘	∘	×	30.15
∘	∘	∘	29.92

**Table 6:** Performance comparison by training amount of the recursive learning stage.

no. of epoch	100	200	300	950
image quality (PSNR)	30.15	29.80	30.01	29.54

**Table 7:** Comparison of denoising performance according to speckle, and salt and pepper noise.

Noise Type	Noise Level	Methods			
		N2B	Recursive	Loss Function	Proposed
speckle	v=0.1	21.85	22.89	24.03	24.33
	v∈[0,0.2]	23.20	24.41	25.16	25.56
salt and pepper	p=0.15	23.30	23.46	23.50	23.71
	p∈[0,0.3]	24.29	24.51	24.56	24.71

**Table 8:** Performance comparison for removing raindrops.

Methods	N2B	Recursive	Loss Function	Proposed
image quality (PSNR)	32.14	33.19	31.90	32.47

## Data Availability

Not applicable.
